# Spaced education activates students in a theoretical radiological science course: a pilot study

**DOI:** 10.1186/1472-6920-12-32

**Published:** 2012-05-23

**Authors:** Emeka Nkenke, Elefterios Vairaktaris, Anne Bauersachs, Stephan Eitner, Alexander Budach, Christian Knipfer, Florian Stelzle

**Affiliations:** 1 Department of Oral and Maxillofacial Surgery, Erlangen University Hospital, Erlangen, Germany; 2 Department of Oral and Maxillofacial Surgery, University of Athens Medical School, Attikon Hospital, Athens, Greece; 3 Department of Prosthodontics, Erlangen University Hospital, Erlangen, Germany; 4 Department of Oral and Maxillofacial Surgery, Erlangen University Hospital, Glueckstr. 11, 91054, Erlangen, Germany

**Keywords:** E-mail, Face-to-face lecture, Learning style, Spaced education, Theoretical radiological science course

## Abstract

**Background:**

The present study aimed at determining if the addition of spaced education to traditional face-to-face lectures increased the time students kept busy with the learning content of a theoretical radiological science course.

**Methods:**

The study comprised two groups of 21 third-year dental students. The students were randomly assigned to a “traditional group” and a “spaced education group”. Both groups followed a traditional face-to-face course. The intervention in the spaced education group was performed in way that these students received e-mails with a delay of 14 days to each face-to-face lecture. These e-mails contained multiple choice questions on the learning content of the lectures. The students returned their answers to the questions also by e-mail. On return they received an additional e-mail that included the correct answers and additional explanatory material.

All students of both groups documented the time they worked on the learning content of the different lectures before a multiple choice exam was held after the completion of the course. All students of both groups completed the TRIL questionnaire (Trierer Inventar zur Lehrevaluation) for the evaluation of courses at university after the completion of the course. The results for the time invested in the learning content and the results of the questionnaire for the two groups were compared using the Mann–Whitney-*U* test.

**Results:**

The spaced education group spent significantly more time (216.2 ± 123.9 min) on keeping busy with the learning content compared to the traditional group (58.4 ± 94.8 min, p < .0005). The spaced education group rated the didactics of the course significantly better than the traditional group (p = .034). The students of the spaced education group also felt that their needs were fulfilled significantly better compared to the traditional group as far as communication with the teacher was concerned (p = .022).

**Conclusions:**

Adding spaced education to a face-to-face theoretical radiological science course activates students in a way that they spend significantly more time on keeping busy with the learning content.

## Background

### Spaced education

Factual knowledge plays a critical role in the development of clinical expertise [1]. Unfortunately, acquired knowledge is often quickly forgotten [2]. Such forgetting raises the important question as to whether the educational process itself might be tailored to improve students’ retention of the curricular material [2]. Spaced education refers to educational programs that are structured to take advantage of the pedagogical benefits of the spacing effect [3]. The spacing effect is the psychological principle that educational encounters that are repeated over time result in more efficient learning [3]. Using standard e-mail to deliver educational content over spaced intervals, several trials have demonstrated the educational efficacy of this methodology as a means to improve overall learning [4,5]. A spaced education item consists of an evaluative component (multiple choice questions) and an educational component (correct answers and detailed explanations of the answers). Upon submitting an answer to a question via e-mail the learner receives the educational component [6].

### Learning styles

Learning styles are characteristic preferences for alternative ways of taking in and processing information [7]. As a consequence, some students prefer to work with concrete information (facts, experimental data), while others are more comfortable with abstractions (theories, symbolic information). Some students are partial to visual presentation of information (pictures, diagrams, flowcharts) and others get more from verbal explanations [8]. Some like to learn by trying things out and seeing and analyzing what happens, and others would rather reflect on things they plan to do and understand as much as they can about them before actually attempting them [8]. In an ideal situation the teaching style matches with the learning styles of the students avoiding students’ demotivation. Learning styles can be determined by the Index of Learning Styles (ILS). It is an online questionnaire designed to assess preferences on four dimensions of a learning style model [9]. The ILS consists of four scales: sensing-intuitive, visual-verbal, active-reflective, and sequential-global. Assessing students’ learning styles helps educators checking if a certain course fulfills the students’ needs.

### Aim of the study

The present study aimed at examining if spaced education

i) activates students in a way that they spend more time on keeping busy with the learning content.

ii) leads to a change in correlation between learning styles and examination results in a theoretical radiological science course.

## Methods

The study was approved by the institutional ethics committee of the University of Erlangen-Nuremberg. 42 third-year dental students were scheduled for the theoretical radiological science course. The learning content comprised radiation physics, X-ray production, X-ray interactions, radiation dose, imaging equipment, radiation protection, image creation, and normal radiological anatomy of the teeth and jaws.

In an introductory explanatory face-to-face session the students were asked to join the study. Participation in the study was optional. Demographic data of the students were documented. The students were randomly assigned to two groups (“traditional group” and “spaced education group”). Each student received a pseudonym as participant of the study. During the introductory session the students had to complete a paper and pen version of the Index of Learning Styles questionnaire (ILS, http://www.engr.ncsu.edu/learningstyles/ilsweb.html). The students indicated their pseudonyms on the questionnaire.

The ILS consists out of 44 items that refer to four scales:

i) Sensing (concrete, practical, oriented towards facts and procedures) or intuitive (conceptual, innovative, oriented toward theories and underlying meanings).

ii) Visual (prefer visual representation of presented material, such as pictures, diagrams, and flow charts) or verbal (prefer written and spoken explanations).

iii) Active (learn by trying things out, enjoy working in groups) or reflective (learn by thinking things through, prefer working alone or with one or two familiar partners).

iv) Sequential (linear thinking process, learn in incremental steps) or global (holistic thinking process, learn in large leaps) [7].

**Figure 1:**
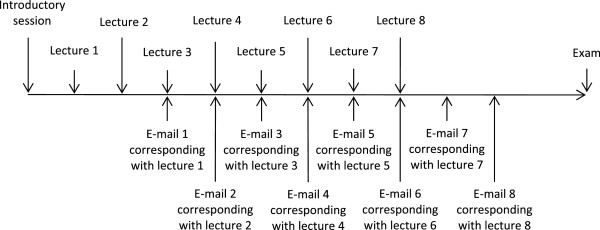
Flowchart of the course of the study.

The answers to the 44 questions could be given in a bipolar fashion (“yes” or “no”). For the determination of the individual learning styles of each single student an online tool was used ( http://www.engr.ncsu.edu/learningstyles/ilsweb.html). One week later, the theoretical radiological science course started. It was delivered as 8 didactic lectures of 45 minutes each. Attendance of the face-to-face lectures was mandatory for both groups of students. The students were asked to document the time they worked on the theoretical radiological science learning content during the duration of the course. While there was no intervention in the traditional group, an intervention was performed in the spaced education group. The intervention was spaced education by the use of e-mails. Following each face-to-face lecture a corresponding e-mail with 3 multiple choice questions on the learning content of the specific lecture was sent out with a delay of 14 days (Figure 1). The students of the spaced education group received an e-mail reminder 3 days after the first e-mail in order to secure the answering of the multiple choice questions within 1 week. When the students had returned their answers to a set of multiple choice questions by e-mail, they received another e-mail that included the correct answers and additional explanatory material. 4 weeks after the last lecture of the theoretical radiological science course the students of both groups had to fill in the TRIL (Trierer Inventar zur Lehrevaluation) questionnaire which is a validated modular German-language questionnaire for the evaluation of courses at university (Table 1) [10]. It comprises 6 topics. Topic 1 (“structure and didactics“) consists of 8 questions that concerned the lecturer’s skills in didactics and structuring of the learning content. Topic 2 deals with the motivational skills of the lecturer consisting of 8 questions. Topic 3 (5 questions) addresses the lecturer’s skills in creating a favorable climate during the course. Topic 4 consisted of 4 questions and asks the students to evaluate practical relevance of the course by providing a connection between theory and practice. Topic 5 subsumes 5 questions on different additional aspects of courses. Topic 6 (“homework”) consisted out of 6 questions that concerned the multiple choice questions sent out by e-mail. This topic had only to be answered by the spaced education group.

**Table 1:** Results of the answers to the TRIL (Trierer Inventar zur Lehrevaluation)

**Question no002E**	**Traditional group**			**Spaced education group**			**p**
	**n**	**Mean value**	**SD**	**n**	**Mean value**	**SD**	
*Topic 1 “structure and didactics“*							
1. The course materials (manuscripts, PowerPoint slides, etc.) provided during the course were helpful for the understanding of the learning content.	21	4.8	1.3	21	4.6	1.0	*.094*
2. Didactic aids (blackboard, flipchart, etc.) were used in an adequate way.	21	4.1	.9	21	4.9	.9	*.034*
3. The lecturer gave short summaries in order to make clear which were the crucial points for the understanding of the topic.	21	3.9	1.3	21	3.7	1.4	*.194*
4. The time management of the lecturer was adequate.	21	5.0	.9	21	4.8	.9	*.334*
5. The learning contents of the single sessions were adapted to the learning targets.	21	4.8	.9	21	4.6	.8	*.450*
6. The course schedule at the beginning of the term gave me a good overview on the learning content.	21	3.3	1.6	21	3.1	1.5	*.777*
7. The course had a reproducible structure.	21	4.5	1.3	21	4.2	1.4	*.060*
8. The course materials were always provided on time.		5.3	1.0		5.3	1.1	*.853*
*Topic 2 “motivational skills of the lecturer“*							
9. The style of speech of the lecturer was fluently and clear.	21	5.4	1.1	21	5.1	1.2	*.650*
10. The lecturer was able to explain difficult learning content in an understandable way.	21	4.1	1.3	21	3.9	1.7	*.631*
11. The lecturer’s speech was acoustically understandable.	21	5.0	1.4	21	5.2	1.0	*.860*
12. The lecturer was able to keep contact to the audience (e.g. by eye-contact).	21	4.9	1.0	21	4.9	1.3	*.915*
13. The lecturer created an inspiring atmosphere.	21	3.7	1.2	21	4.1	1.5	*.290*
14. The lecturer was able to deal with disturbances (technical problems, noisiness, etc.).	21	4.3	1.2	21	4.9	1.3	*.756*
15. It was easy for me to remain concentrated during the course.	21	3.7	1.2	21	3.7	1.4	*.553*
16. I was inspired to follow the train of thoughts during the course.	21	4.0	1.1	21	3.8	1.7	*.087*
*Topic 3 “the lecturer’s skills in creating a favourable climate”*							
17. The lecturer stopped discussions at the right point of time.	21	4.7	1.0	21	4.4	1.3	*.070*
18. The lecturer treated the students friendly and was open-minded.	21	5.4	.8	21	5.5	.8	*.563*
19. The lecturer allowed asking questions that concerned the learning content and answered them adequately.	21	5.5	.6	21	5.4	.7	*.672*
20. The students received the possibility to give contributions to the course.	21	5.4	1.0	21	5.0	1.1	*.146*
21. The lecturer was able to fulfill needs expressed by the students concerning content, structure and organization of the course.	21	3.6	1.4	21	4.6	1.4	*.022*
*Topic 4 “practical relevance of the course”*							
22. During the course the relation between theoretical knowledge and practical application demonstrated.	21	4.9	1.0	21	4.6	1.2	*.400*
23. The learning content of the course was adequately illustrated by practical examples (case studies, clinical applications, etc.).	21	4.8	1.1	21	4.4	1.6	*.505*
24. I was inspired to deal with the learning content critically.	21	4.0	1.2	21	3.8	1.6	*.456*
25. The practical relevance of the learning content should have been highlighted even more intensively.	21	3.3	1.4	21	2.7	1.4	*.422*
*Topic 5 “questions on different additional aspects”*							
26. The availability of the lecturer at other occasions than the lecture was satisfying.	21	5.3	1.2	21	5.6	.7	*.676*
27. Even at other occasions than the lectures the lecturer answered my questions in an adequate way.	21	5.0	.9	21	5.1	1.2	*.447*
28. I prepared myself for the lectures on a regular basis (e.g. by reading of additional literature).	21	1.6	.7	21	1.8	.8	*.762*
29. I did follow-up course work on a regular basis (e.g. by discussion with other students or by reading of additional literature).	21	3.2	1.2	21	3.6	1.1	*.797*
30. The degree of difficulty of the course was 1 = too low, 2 = low, 3 = adequate, 4 = high, 5 = too high.	21	3.3	.5	21	3.3	.6	*.833*
*Topic 6 “homework”*							
31. The level of difficulty of the homework was adequate.	/	/	/	21	4.5	1.2	*/*
32. The homework had a good training effect.	/	/	/	21	4.0	1.4	*/*
33. The homework was an adequate way of preparing for lectures and following up lectures.	/	/	/	21	3.7	1.4	*/*
34. The homework was worded in an adequate way.	/	/	/	21	5.3	.8	*/*
35. The homework improved my understanding of the learning material.	/	/	/	21	4.1	1.3	*/*
36. The amount of homework was adequate.	/	/	/	21	5.5	.8	*/*

Answers could be chosen between 1 and 6 (1 = I totally disagree, 6 = I totally agree). For Question 30 possible answers were 1 = too low, 2 = low, 3 = adequate, 4 = high and 5 = too high.

The spaced education group also had to answer 10 additional questions that were put together by the authors. These questions concerned the students’ attitude towards receiving and answering e-mails on the learning content of the course (Table 2). For all questions the answers could be given on a scale from 1 to 6 (1 = I totally disagree, 6 = I totally agree).

**Table 2:** Data of the answers given by the spaced education group to the questionnaire on students’ attitudes towards spaced education

**Question no.**	**n**	**Mean value**	**SD**
1. E-mails sent to me with questions on the course’s learning content helped me to keep me busy working on the learning content on a continuous basis.	21	3.6	1.4
2. Receiving e-mails with answers to the questions answered previously was an additional help.	21	4.3	1.7
3. The questions sent by e-mail helped me to get a deeper insight in the learning content.	21	4.0	1.7
4. The e-mails that contained questions regarding the learning content did negatively intrude my private life.	21	1.4	1.0
5. The amount of learning content that I had to work through in order to answer the questions sent by e-mail was adequate.	21	4.9	1.1
6. I would have preferred receiving more questions on the learning content by e-mail.	21	2.4	1.6
7. Answering the questions that I received by e-mail kept me from working on the content of other courses.	21	2.2	1.7
8. I would have preferred receiving questions on the learning content directly after the single lectures.	21	2.7	1.8
9. It would have been sufficient to just show the multiple choice questions on the final slide of each lecture.	21	2.6	.9
10. I do not see any need to change the didactical concept of the course.	21	2.7	1.1

Answers could be chosen between 1 and 6 (1 = I totally disagree, 6 = I totally agree).

In the same session an exam on the theoretical radiological science course was held for both groups. The students had to answer 20 multiple choice questions. These questions covered the same topics as the multiple choice questions sent out by e-mail to the spaced education group, but they were not identical.

### Statistical analysis

Mean values are given with standard deviations. The *χ*^2^ test was used to test if there was a statistically significant difference in the gender distribution between the two groups. For comparison of continuous variables in unpaired samples the Mann–Whitney-*U* test was adopted.

Cronbach’s α analysis was carried out to assess reliability of the exam that adopted multiple choice questions. α-values of .7 or higher are in the acceptable range recommended by the literature [11]. α-values above .8 reflect a high reliability.

In order to assess the correlation between learning styles and examination results Spearman’s rho was calculated. P-values less than or equal to .05 were considered significant. All calculations were made using SPSS Version 14.0 for Windows (SPSS, Chicago, USA).

## Results

All students chose to join the study. The analysis of the demographic data revealed that there was no statistically significant difference in age between the two groups (24.7 ± 2.2 years in the traditional group, 24.3 ± 2.8 years in the spaced education group, p = .837). In both groups there were more females than males (15 females and 6 males in the traditional group, 16 females and 5 males in the spaced education group). However, the distribution in gender did not differ statistically significantly for the two groups (p = .726).

All face-to-face lectures took place as scheduled. No technical problems were encountered with sending and receiving of the e-mails. All questionnaires were filled in adequately before and after the theoretical radiological science course.

During the theoretical radiological science course the spaced education group spent significantly more time (216.2 ± 123.9 min) on keeping busy with the learning content compared to the traditional group (58.4 ± 94.8 min, p < .0005).

The results for the TRIL questionnaire are given in Table 1. The members of the spaced education group rated the didactics of the course significantly better than the members of the traditional group (Q2, p = .034). The spaced education group also felt that their needs were fulfilled significantly better compared to the traditional group as far as the communication with the teacher was concerned (Q21, p = .022). The answers of the spaced education group to the TRIL topic 6 “homework” showed that the students considered working on the multiple choice questions sent out by e-mail an adequate way of improving their knowledge in the field (Table 1).

The answers to the additional 10 questions put together by the authors revealed that the students of the spaced education group appreciated the e-mails because they helped them working continuously of the learning content of the course (Table 2). The students did not consider the sending of e-mails on the course content an intrusion of their private lives. A time span of two weeks between a face-to-face lecture and the sending of corresponding e-mails was considered adequate by the students. The answers to the questions showed that the students would not have preferred receiving the multiple choice question directly after the single lectures or at the end of each presentation.

The scores for the learning styles for both groups are given in Table 3. There was no statistically significant difference in the distribution of the different learning styles between the traditional group and the spaced education group. For the exam a Cronbach’s α-value of .72 reveals was calculated. This value reflects an acceptable reliability of the exam.

**Table 3:** Index of Learning Styles results

**Learning styles**	**Traditional group**			**Spaced education group**			**p**
	**n**	**Mean value**	**SD**	**n**	**Mean value**	**SD**	
Active/reflective	21	Active 1.2	2.1	21	Active 2.5	1.8	*.326*
Sensing/intuitive	21	Sensing 4.7	1.9	21	Sensing 3.3	2.7	*.452*
Visual/verbal	21	Visual 6.6	2.0	21	Visual 6.9	2.1	*.594*
Sequential/global	21	Sequential 1.3	2.2	21	Global 1.3	2.4	*.194*

For each of the two dimensions of each learning style scale reached to a maximum value of 11. Scores equal to or below 3 indicate that the learning style is balanced on the two dimensions. Scores larger than 3 indicate that there is a preference for one dimension of the learning style.

For the traditional group there was a significant correlation between an active/reflective learning style and the examination results with improved results for an active learning style (p = .012) and a sequential/global learning style and the examination results with improved results for a sequential learning style (p = .013). Sensing/intuitive (p = .849) and visual/verbal (p = .721) learning styles did not show statistically significant correlations to the examination results. For the spaced education group there were no significant correlations between learning styles and examination results (active/reflective: p = .165, sequential/global: p = .784, sensing/intuitive: p = .826, visual/verbal: p = .932).

## Discussion

Spaced education is an evidence-based form of education that has been demonstrated to improve knowledge acquisition and to boost knowledge retention [12]. Especially, it enhances the effect of traditional face-to-face lectures [12]. In the past it has already been mentioned that spaced education is well accepted by learners [13,14].

The present study aimed at examining if spaced education

i) activates students in a way that they spend more time on keeping busy with the learning content.

ii) leads to a change in correlation between learning styles and examination results in a theoretical radiological science course.

In the present study students rated the didactics of the course significantly better, when spaced education was used. It has been described previously that students who received online material in addition to face-to-face lectures reported a better understanding of the learning content [15]. Comparable results have been found in the present study. The students of the spaced education group were positive about receiving multiple choice questions via e-mail and getting explanations on the correct answers via e-mail later on (Table 2).

The way spaced education was delivered in the present study seems to be a form of communication that students appreciate. This aspect is reflected by the answers to Q21 of the TRIL questionnaire (Table 1). The students of the spaced education group rated the ability of the teacher to fulfill their needs concerning communication significantly better compared to the students of the traditional group. Although learning was extended to their spare time, the students of the spaced education group were positive about this form of teaching and learning. The students denied that the e-mails sent to them on the course content intruded their private lives in a negative way (Table 2). Instead, theses e-mails offered a new possibility to structure learning with the consequence that the members of the spaced education group spent significantly more time on keeping busy with the learning content compared to their counterparts of the traditional group. In the present study spaced education led to a relevant activation of the learners.

Several different time intervals have been proposed for the delivery of spaced education items [4]. In the present study a time interval of two weeks was chosen between the lectures and e-mails containing the spaced education items. The answers to the questionnaire put together by the authors reveal that the time interval was well accepted by the students (Table 2). They did not go for a shorter time interval or direct delivery of the multiple choice questions at the end of each presentation. Today’s students seem to accept the deliverance of spaced education items as a way of providing additional information on lectures.

It has been show previously that face-to-face lectures favor learners with specific learning styles [16,17]. However, one learning style is neither preferable nor inferior to another, but is simple different with different characteristic strengths and weaknesses [16,17]. Therefore, courses should address all learning styles. It is well known that greater learning may occur when teaching styles match learning styles than when they are mismatched [8]. The combination of face-to-face lectures with spaced education may have the desired effect. The tendency that students with specific learning styles achieved better examination results was leveled. To the best of our knowledge, this effect of spaced education has not been described in the current literature before. Combining face-to-face lectures with spaced education seems to improve the chances of benefiting from a course for all students whose learning style is not completely suited for face-to-face lectures.

The present study is limited by different aspects. First of all, the number of participants is low. As a consequence, the results have to be confirmed in larger student populations.

It is difficult to say if the Hawthorne effect influenced the results of the study. It addresses an increase of productivity in the control group that did not receive an intervention as a consequence of increased attention of the observer of the study to this group [18]. Both groups documented the time they spent on keeping busy with the learning content. The spaced education group invested significantly more time. As a consequence it can be assumed that the Hawthorne effect did not play a major role in the present study.

Another limitation of the study is that in the examination only a limited number of multiple choice questions was used. However, the Cronbach’s α-value of .72 reveals that the test had an acceptable reliability. Still efforts have to be made to increase Cronbach’s α-value above .8 in future trials in order to reach a higher level of reliability [11].

## Conclusions

Adding spaced education to a face-to-face theoretical radiological science course significantly activates students in a way that they spend more time on keeping busy with the learning content. Moreover, combining spaced education with face-to-face lectures gives a view to a more balanced way of education that does not prefer specific learning styles.

## Competing interests

The authors declare that they have no competing interests.

## Authors’ contributions

EN and FS made substantial contributions to conception and study design and wrote the manuscript. EV and SE made substantial contribution to conception and study design. AB1, AB2 and CK carried out the data collection and performed the statistical analysis. All authors interpreted the results, drafted the manuscript and read and approved the final manuscript.

## Pre-publication history

The pre-publication history for this paper can be accessed here:

http://www.biomedcentral.com/1472-6920/12/32/prepub
